# Sustainable Valorization of *Sambucus nigra* L. Berries: From Crop Biodiversity to Nutritional Value of Juice and Pomace

**DOI:** 10.3390/foods11010104

**Published:** 2021-12-31

**Authors:** Carina Pedrosa Costa, Samuel Patinha, Alisa Rudnitskaya, Sónia A. O. Santos, Armando J. D. Silvestre, Sílvia M. Rocha

**Affiliations:** 1 LAQV-REQUIMTE & Department of Chemistry, Campus Universitário Santiago, University of Aveiro, 3810-193 Aveiro, Portugal; carina.pedrosa@ua.pt; 2 CICECO-Aveiro Institute of Materials & Department of Chemistry, Campus Universitário Santiago, University of Aveiro, 3810-193 Aveiro, Portugal; jsamuelpatinha@ua.pt (S.P.); santos.sonia@ua.pt (S.A.O.S.); armsil@ua.pt (A.J.D.S.); 3 CESAM & Department of Chemistry, Campus Universitário Santiago, University of Aveiro, 3810-193 Aveiro, Portugal; alisa@ua.pt

**Keywords:** *Sambucus nigra* L., elderberry, natural products, maturity, juice, berry pomace, proximate composition, nutritional value, food products

## Abstract

Improvement of dietary and ecological biodiversity, namely by exploring autochthonous varieties, is a key point to the construction of a more sustainable food system and planetary health. However, the environmental sustainability continues to face huge challenges, reflecting the importance of achieving a better understanding about the functional role of biodiversity in ecosystems. Thus, the main objective of this research is to contribute to the sustainable valorization of *Sambucus nigra* L. berries through a comprehensive approach to evaluate the effects of elderberry’s cultivar, harvest year, and plantation field on the physicochemical berry composition. Moreover, the nutritional value of elderberry juice and respective dried pomace was determined. This complementary information is of huge utility for the rational and, as much as possible, integral use of elderberries. The harvest year, followed by field and the interaction of harvest × field, accounted for the highest impact on the berry’s physicochemical parameters, indicating the importance of the combined impact of the macro- and mesoclimate conditions on plant metabolism. Elderberry juice and dried pomace are a good source of carbohydrates (ca. 12 and 82%, respectively) and have low amounts of fat (≤2.5%), making them low-energy foods. Dried pomace may also represent a potential alternative source of vegetal protein (ca. 6%).

## 1. Introduction

According to the Food and Agriculture Organization of the United Nations (FAO), there are over 50,000 edible plants; however, only 60% of human energy intake comes from just three plant species: rice, maize, and wheat. In addition, this trend is expected to increase by 33% until 2050 [1]. The lack of dietary diversity impairs human health and environment and provokes an overproduction of some foods and underproduction of others [2]. Moreover, the World Health Organization (WHO) reported that it is common to find undernutrition and obesity coexisting within the same country, the same community, and the same household. Nowadays, other challenges must be overcome regarding the continuous growing of world population, and the requirements to produce enough food to meet the needs of the human population, and the limited natural resources already under pressure due to expected climatic changes. Furthermore, the food industries, namely agri-industry, are also increasingly focused on meeting sustainability requirements and reorganizing their entire value chain to achieve that goal. In fact, it is important to promote a circular economy strategy focusing on positive society-wide benefits. This concept entails serious concerns about the rational and integral use of natural resources, as much as possible, contributing to the reduction in byproducts. The valorization of byproducts should also be a central concern, notably through their exploitation as a source of value-added food ingredients that might be introduced sustainably into the food chain [3,4,5]. In general, food processing generates a large amount of byproducts that now represent ca. 33% of total food products, and a multitude of projects have been developed to valorize this low-cost source of bioactive compounds [3].

Moreover, a balanced diet is also a key step for the transition to a more sustainable food system and planetary health. In fact, from an environmental point of view, diversification of cropping systems achieves the main goal of increased sustainability. On the other hand, from a health point of view, it is projected that improving well-being and a healthy diet with a sustainable food production system can prevent 11 million premature adult deaths per year [3]. The dietary nutrients are essential to the maintenance of body functions, with the macronutrients providing the major sources of energy, while micronutrients play a central role in metabolism, for example, through providing essential cofactors for enzymatic functions, as well as the maintenance of specific tissue function [6].

Plant-based products, including the agri-food byproducts, have been recognized as a natural source of nutrients and bioactive compounds considered as health and well-being promoters [7,8,9,10], and particular attention must be given to the species that are not part of the large commercial circuits. In this context, elderberry (*Sambucus nigra* L.) was identified as an attractive crop as it has been reported as a source of dietary phytochemicals, namely fiber, vitamins, phenolic compounds, amino acids, unsaturated fatty acids, and minerals, among others [11,12,13,14,15]. Elderberry has been used for centuries as a source of food additives and nutraceuticals. The potential human health benefits of these berries include antiviral, anti-inflammatory, antioxidant, anticarcinogenic, immune-stimulation, and antibacterial properties [16,17,18,19,20]. Recent study reported the potentials of natural bioactive compounds against obesity, and phenolic compounds, in particular, were identified to play a crucial role in tackling obesity via regulating appetite [21].

Elderberries are used in the food industry to produce mainly juices and juice concentrates, but a wide range of other products, such as pies, jellies, jams, ice creams, and yogurts, have also been produced incorporating this natural product [22,23,24]. Moreover, food and pharmaceutical industries have shown a great interest for elderberry extracts as a source of antioxidants and colorant compounds [11,14,25]. The production of juices generates a significant amount of wastes, namely pomace that can be exploited for that vein, contributing to the reduction in food waste and providing important environmental and economic benefits [12]. Despite the potential value of pomace, from the point of view of industrial applications, it remains unexplored. To improve further developments, detailed knowledge of its composition and physicochemical properties is essential. 

Berry dried pomaces showed acceptable color stability, with additional benefits regarding phenolic compounds and dietary fiber content [26]. Particularly, the elderberry pomace showed promising techno-functional properties for possible food applications, such as the particle size distribution, bulk density, sedimentation velocity, and swelling capacity [26]. In addition, the consumers are progressively pressing the food industry to provide new food products with the minimum artificial additives. Namely, due to the frequently associated side effects to artificial colorants, such as allergic reactions and toxicity, among others, the food industry is increasingly looking for natural colorants as effective replacements for artificial ones [13].

In summary, elderberries and byproducts resultant for their industrial processing may be considered as matrices with high potential to fulfil the current challenges associated to the construction of a more sustainable food system and planetary health. In fact, FAO suggests several actions to improve dietary and ecological biodiversity, for instance, diversifying the diet with new crops and previously forgotten foods, namely by exploring autochthonous varieties [1,2]. These strategies can also have a very significant impact on the economy of local and family communities. In Portugal, *Sambucus nigra* L. plantation is almost exclusively located at Varosa Valley (Northern Portugal) and ‘Sabugueiro’, ‘Sabugueira’, and ‘Bastardeira’ are the main elderberry cultivars cultivated in this region [27,28,29]. The elderberry crop has shown a great capacity to adapt to this region, and previous study reported that the chemical composition of elderberries may depend on the combination of several intrinsic and extrinsic factors [8]. Thus, particular attention should be devoted to the spatial and temporal interactions between autochthonous varieties, clime, and field conditions, such as soil type, orientation of the lines, age of the plantation, irrigation, and planting density, among others. The environmental sustainability continues to face huge challenges, meaning that it is important to achieve a better understanding about the functional role of biodiversity in ecosystems.

The main objective of the present study is to contribute to the sustainable valorization of *Sambucus nigra* L. berries through a comprehensive approach to evaluate the effects of elderberry’s cultivar, harvest year, and plantation field on the physicochemical berry composition. Thus, pH, titratable acidity, total soluble solids content, total phenolic content, and antioxidant activity, determined by DPPH method, were followed throughout ripening using three *S. nigra* cultivars produced in three fields over three consecutive years. As scarce information is available about the nutritional value of elderberry juice and respective dried pomace, the proximate composition of these products, which includes moisture, ash, lipid, protein, and carbohydrate contents, were determined. Energetic value, mineral, and vitamin B6 composition were also evaluated. This complementary information is of huge utility for the rational and, as much as possible, integral use of elderberries.

## 2. Materials and Methods

### 2.1. Field and Weather Characteristics and Sampling

#### 2.1.1. Field Characteristics

**Figure 1:**
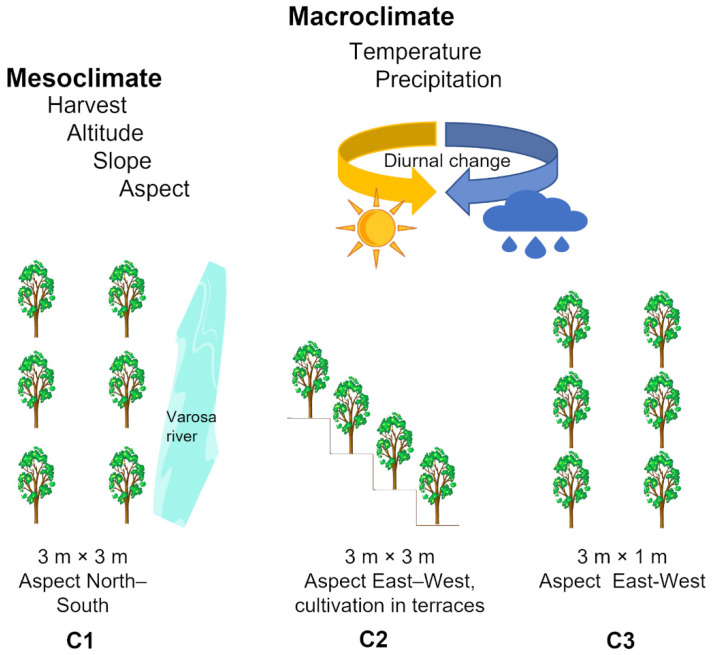
Schematic representation of the three plantation fields (Varosa (C1), Valverde (C2), and São João de Tarouca (C3)) where the three cultivars of *Sambucus nigra* L. ‘Sabugueira’, ‘Sabugueiro’, and ‘Bastardeira’ were grown. Both the macroclimate in the Varosa Valley region and the mesoclimate conditions in each field may impact the ripening process.

Elderberry from *Sambucus nigra* L. cultivars ‘Sabugueira’, ‘Sabugueiro’, and ‘Bastardeira’ from three consecutive harvesting periods (2018–2020), were collected through ripening, in three fields located at Varosa Valley (Portugal): Varosa (C1) (41°04′11″ N; −7°45′40.1″ W), Valverde (C2) (41°04′19″ N; −7°44′39.8″ W), and São João de Tarouca (C3) (40°59′42.9″ N; −7°44′43.8″ W). The main characteristics of the fields under study are summarized in Table 1. The three fields have plants from the three cultivars, and the plant-to-plant distance was 3 m × 3 m for C1 and C2, and 3 m × 1 m for C3 (Figure 1).

The C1 field is confined within a valley (120 m of altitude), protected from winds, and the Varosa River borders the northeast field. The C2 field is located at an altitude of 640 to 700 m, and the elderberry trees are cultivated on the terraces, in which each row is on a terrace, and the C3 field is located at an altitude of 570 m. All soils contain a low organic matter content, medium to light texture, acidic pH (4.7–5.3), and high potassium content (≥70 ppm K_2_O). 

#### 2.1.2. Harvest Weather Conditions

The harvest year weather information (mean precipitation and mean temperature) for a 10-year period (2010–2020) was obtained from the local meteorological station (type: EMA I climatologic station, number 560), from Instituto Português do Mar e da Atmosfera (IPMA, IP, https://www.ipma.pt/pt/, accessed on 10 May 2021), located in the municipality of Viseu (Portugal).

**Figure 2:**
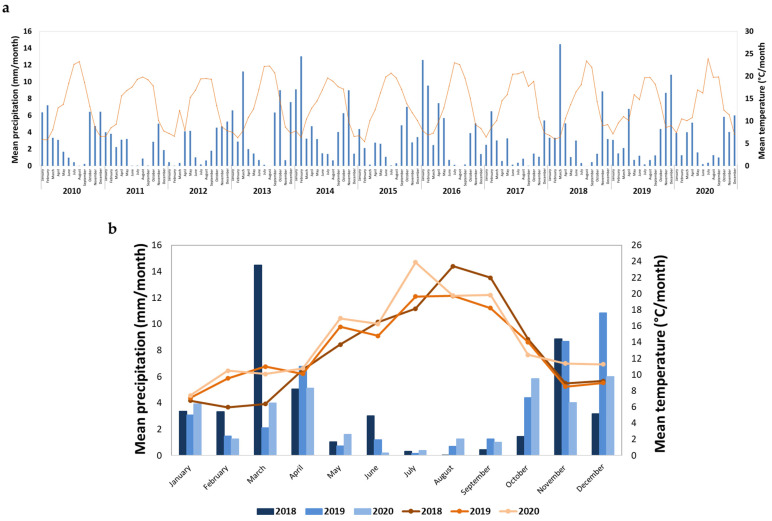
Meteorological data available for Varosa Valley, expressed as mean precipitation (blue colors) and temperature (orange colors); (**a**) data from the last decade (2010–2020) and (**b**) zoom of the three harvests under study (2018–2020).

As can be observed in Figure 2, the mean precipitation and mean temperature ranges over the years 2018 to 2020 are within the ranges observed in the last decade. The maximum precipitation was observed in the period November to December, and in March for year 2018. Although the occurrence of moderate precipitation in spring is common, in 2018, an unusual precipitation was observed (Figure 2a). Furthermore, 2018 exhibited an unusual fresh spring, while 2019 and 2020 exhibited moderate temperatures during spring (Figure 2a), which were considered suitable for the maturation process. 

#### 2.1.3. Sampling

*S. nigra* plants were marked in three fields and the berries were collected from the selected plants over the 3 harvests through ripening. Maturity stages were defined based on the total soluble solids (expressed as °Brix), pH, titratable acidity, and the homogeneous pigmentation of the elderberries (to be harvested, 75% of the berries must be dark violet). The maturity stages selected were defined as containing berries at the early stage of maturity—approximately 30% of berries exhibited the final color (EM); containing almost mature berries—approximately 70% of berries exhibited the final color (AM); and containing fully mature berries (FM).

For the preparation of the juice and pomace powder ca. 10 kg of fully mature berries (mixture prepared from 3 cultivars, simulating what happens during the collection of the berries to be delivered to suppliers, in which there is no separation of cultivars) were collected from the 2020 harvest. After being collected, the samples were transported immediately under refrigeration (*ca.* 4 °C) to the laboratory.

### 2.2. Eldeberry Physicochemical Parameter Determination

#### 2.2.1. pH, °Brix, and Titratable Acidity

First, the elderberry samples were weighed and crushed, the corresponding juice suspensions were centrifuged at 10,000 rpm for 15 min at 4 °C, and the resulting juice filtered through sterile gauze to remove residual impurities, such as seeds, to obtain clarified juice samples. 

The pH of juice samples was measured using a pH meter (micropH 2002, Crison, Barcelona, Spain). The total soluble solids content (TSS) was established through Brix degree (°Brix) measurement using a refractometer (A. KRÜSS Optronic refractometer, Hamburg, Germany). The titratable acidity (TA) was measured by titrimetry using NaOH 0.02 and 0.04 M (Panreac, Barcelona, Spain), and pH was measured using a pH meter. All measurements were made with 5 replicates, each one corresponding to an independent aliquot.

#### 2.2.2. Total Phenolic Content

The total phenolic content (TPC) was determined using Folin–Ciocalteu assay, using a previously implemented method from Touati et al. [30] adapted to 96-well plates. Briefly, the elderberry juice was diluted 200 times and 30 µL of diluted sample was put into the 96-well plate. For the blank, 30 µL of water was substituted for the sample. For the calibration curve, 30 µL of the gallic acid standard solutions (12.5–200 mg/L) were placed in the 96-well plate. Then, 150 µL of Folin–Ciocalteu reagent was added to the 96-well plate and adding 120 µL of NaCO_3_ (75 g/L) solution. After 5 min of incubation in the dark at 50°C, the absorbance was determined at 765 nm using a UV–visV-530 spectrophotometer (Jasco, Tokyo, Japan). The TPC was expressed as grams of gallic acid equivalents (GAE) per liter of elderberry juice (g GAE/L juice). All measurements were made with 5 replicates, each one corresponding to an independent aliquot.

#### 2.2.3. Antioxidant Activity

Antioxidant activity was determined based on the 2,2-diphenyl-1-picrylhydrazyl (DPPH) assay, adapted from Touati et al. [30] to 96-well plates. The elderberry juice was diluted 200-fold and 25 µL of diluted sample was put into the 96-well plate. For the blank, 25 µL of water was used instead of the sample. For the calibration curve, 25 µL of Trolox standard solutions (25–800 µmol/L) were placed in the 96-well plate. Then, 275 µL of 2,2-diphenyl-1-picrylhydrazyl (DPPH^●^) (125 mM in MeOH) was added to the 96-well plate. After 30 min of incubation in the dark at room temperature, the absorbance was determined at 517 nm using a UV–visV-530 spectrophotometer (Jasco, Tokyo, Japan). The antioxidant activity was expressed as mmol of Trolox equivalents per liter of elderberry juice (mmol TE/L juice). All measurements were made with 5 replicates, each one corresponding to an independent aliquot.

### 2.3. Eldeberry Products

#### 2.3.1. Preparation of Elderberry Juice and Pomace Powder

The elderberries were weighed, and then crushed and pressed, and the suspension was centrifuged at 10,000 rpm for 15 min at 4 °C. The juice was filtered through sterile gauze to remove residual impurities and obtain a clarified juice. The remaining pomace was freeze-dried using VirTis BenchTop K (SP Industries, Warminster, PA, NY, USA), and, after this, seeds were separated from the solid residue for further processing into pomace powder. The pomace powder was prepared with a PULVERISETTE 11 (Fritsch, Idar-Oberstein, Germany) at 14,000 rpm for 60 s.

#### 2.3.2. Determination of Nutritional Value

The nutritional composition of the elderberry juice and pomace powder was estimated using standard methods—AOAC analytical procedures [31]. The total carbohydrates were calculated by difference and the energetic value was calculated according to the Regulation (EC) Number 1169/2011 of The European Parliament and of the Council as follows: energy (kcal) = 4 × (g protein + g carbohydrate) + 9 × (g fat). The total fat determination was performed with an acid hydrolysis method, followed by extraction using a Soxhlet apparatus (Soxtec™ 2050) for 1 h 30 min with petroleum ether (40–60 °C) as the extraction solvent. The residue obtained was dried for 1 h 30 min at 102 ± 2 °C, until constant weight, followed by gas chromatography analysis according to method 983.23 of the AOAC International. For protein quantification, each sample was analyzed in duplicate for total nitrogen by the Kjeldahl method [32] in combination with a copper catalyst using a block digestion system Foss Tecator 2006 Digestor (Höganäs, Sweden) and a Foss 2800 Kjeltec Auto Distillation unit (Foss Tecator, Hilleroed, Denmark). The protein content was calculated by using 6.25 conversion factor, according to method 981.10 of the AOAC International. The content of total dietary fiber (TDF) was determined by the enzymatic–gravimetric method (AOAC, 2000) in a Fibertec™ 1023 (includes a WB 1024 Water bath and a Filtration Module). Samples were weighed in duplicate (0.5 g) and enzymatic digestion with α amylase, protease, and amyloglucosidase was applied. A duplicate blank assay was performed using the same procedure as the digested sample.

Moisture content was determined by the gravimetric method, using a dry air oven from Heraeus Instruments, Hanau, Germany, at 102 ± 2 °C during 2 h, using 5 g of sample until constant weight. Total ash analysis was carried out in a muffle furnace M110 (Heraeus Instruments, Hanau, Germany) at 525 ± 25 °C for 20 h, using 5 g of sample until constant weight, according to method 940.26 of the AOAC International.

Quantification of Ca, Mg, and Fe was performed by inductive coupled plasma optical emission spectroscopy (ICP-OES) (Thermo iCAP 6000 series, with radial and axial configuration, Waltham, MA USA) following the 929.07 and 931.10 of the AOAC International. The quantification of Se was carried out by inductively coupled plasma mass spectrometry (ICP-MS) (Thermo Xseries II, Waltham, MA USA).

Quantification of vitamin B6 was performed by reverse-phase high-performance liquid chromatography (HPLC) with fluorescence detection (Waters Alliance HPLC System 2695 with Multi-Wavelength Fluorescence Detector 2475) according to method 961.15 of the AOAC International.

### 2.4. Data Processing

Effects of elderberry’s cultivar, harvest year, and plantation field on the chemical berry composition was evaluated using analysis of variance–simultaneous component analysis (ASCA). The dataset comprised 135 samples of three varieties from three fields, three harvests (2018–2020), and five independent replicates. The significance of the effects of three main factors and their interaction was evaluated using a permutation test with 2000 permutations. Percentage of the variance explained by each factor or interaction was used as a quality-of-fit criterion [33,34]. ASCA and a permutation test were implemented in Matlab^®^ R2020b (Mathworks, Inc., Natick, MA, USA) using the algorithms already described [34,35].

## 3. Results and Discussion

### 3.1. Spacial and Temporal Variability of Eldeberry Physicochemical Composition

The parameters currently used to define the technological maturity stage of the elderberries are pH and titratable acidity (to estimate acidity and, indirectly, taste and microbial stability), and total soluble solids (TSS) content, which expresses the soluble sugar content and helps to delineate harvesting time. The commercial value of the berries is usually associated to these parameters’ values. In this study, these parameters were used to evaluate physicochemical composition of elderberries, considering three cultivars collected through three consecutive harvesting periods (2018–2020) and in three fields (Table 2). As elderberries have been reported as a valuable source of phenolic compounds and antioxidants [12,15,25], the TPC and antioxidant activity, determined by DPPH method, were also followed throughout ripening (Table 3).

For all the samples under study, the pH and total soluble solids values tended to increase through ripening, ranging from 3.43 to 5.02, and 5.0 to 20.4 °brix (Table 2), respectively, which is in line with expected results [36,37]. These results also reinforce that the elderberries from Portuguese cultivars present a higher TSS content than that reported for other European cultivars, for instance, ‘Haschberg’ cultivar, which is the major cultivar cultivated in northern Europe and presents a TSS content ca. 12 for ripe berries [27,38]. These differences may be explained by the specific edaphoclimatic conditions of mainland Portugal, with average temperatures higher than those occurring in central and northern Europe and presenting a higher number of hours of daily sun exposure. Considering the results within each field, it was observed that the ‘Sabugueira’ cultivar tended to present higher TSS content when compared to ‘Bastardeira’ and ‘Sabugueiro’, which are in accordance with previous results reported for other fields on the same region [37].

The titratable acidity values decreased along ripening, ranging from 1.33 to 0.29 g of citric acid/L juice (Table 2). These values are within those reported in the literature for these cultivars [37] and other cultivars from *S. nigra* grown in other countries (titratable acidity from 0.48 to 1.43 g of citric acid/L juice) [36,38].

Table 3 shows that the total phenolic content and antioxidant activity increase during ripening for all fields, cultivars, and harvest years. The lowest total phenolic content was observed for EM elderberries in C1 field (1.35 mg GAE/L juice) and the highest value was observed for berries collected in field C2 (22.37 g GAE/L juice), both for ‘Sabugueira’ cultivar and the 2018 harvest. The increase in anthocyanins and other phenolic compound content during ripening contributes to the observed increase in the antioxidant activity [28,39,40,41]. The anthocyanins, of which cyanidin 3-glucoside, cyanidin 3-sambubioside, cyanidin 3-sambubioside-5-glucoside, and cyanidin 3,5-diglucoside are the most abundant, and cyanidin 3-rutinoside, pelargonidin 3-glucoside, and pelargonidin 3-sambubioside are present in smaller amounts, represents the main group of elderberry phenolic components [12,39]. Apart from the ripening stage, various factors may influence the total phenolic content, including plant genotype, field, and weather conditions in the harvest year. For instance, Mikulic-Petkovsek et al. [41] reported varying levels of total phenolics in wild and cultivated berries. Another study reported that cultivated elderberries had a higher content of phenolic compounds compared to the wild ones [42]. Furthermore, previous study reported that elderberry ‘Haschberg’ and ‘Korsor’ cultivars exhibited distinct anthocyanins profiles, with total phenolics content varying significatively depending of harvest [38]. The variation in both antioxidant activity and total phenolic content follows the same trend (Table 3), and a positive association was observed between these parameters and the TSS determined during ripening (Figure S1).

As the main objective of this work is to understand spatial (across the fields) and temporal (across the harvest) variability of elderberry physicochemical composition, a statistical approach was applied for evaluation of the potential effects of harvest, field characteristics, and elderberry’s cultivar (Table 4). Since the ultimate objective is to assess the variability observed for ripe berries, only berries at FM stage were considered here. Descriptive statistics for the physicochemical parameters for the berries of three cultivars and from three fields and three harvests are shown in Table 5 and Figures S2 and S3. As all three cultivars were present in all fields in similar proportions, the median values of physicochemical parameters per field (for all years and cultivars), per year (for all field and cultivars), and per cultivar (for all years and fields) were calculated. In summary, ripe berries indicate that TSS median values ranged between 13 and 16 °Brix, titratable acidity between 0.38 and 0.59 g citric acid/L juice, and pH between 4.40 and 4.87. The median total phenolic content ranged between 9.84 and 18.88 g GAE/L juice and the antioxidant activity ranged between 54.04 and 80.28 mmol TE/L juice (Table 5 and Figures S2 and S3).

Statistical significance of the effects of cultivar, field, and harvest year on the physicochemical parameters of the ripe berries was evaluated using ASCA. The resulting *p*-values are shown in Table 4.

According to Table 4, effects of all three factors were statistically significant. The harvest presents the largest sources of the variability in data (*p*-value < 0.0005), explaining 35.1% of total dataset variance. The field effect (*p*-value < 0.0005) accounted for 18.3% of the total set variance, while the cultivar accounted for 5.7% of the total set variance (*p* < 0.05). All three interaction effects, harvest x field; field x cultivar; and harvest x cultivar, were also statistically significant (*p* < 0.05).

**Figure 3:**
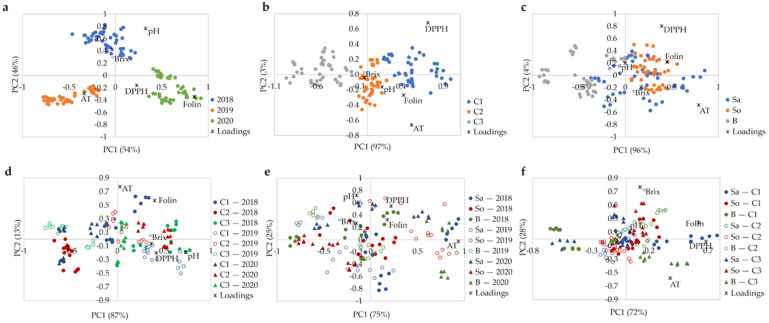
ASCA biplot for (**a**) harvest, (**b**) field, (**c**) cultivar, (**d**) harvest × field, (**e**) harvest × cultivar, (**f**) field × cultivar factors obtained for ripe elderberries (three cultivars collected on three fields for three consecutive harvests were considered); significance test reported in Table 4.

According to the meteorological data, the mean precipitation and mean temperature in the years 2018 to 2020 were within typical ranges observed in the last decade, while the year 2019 was the freshest of the three (Figure 2b). Furthermore, in 2018, higher precipitation and lower temperature during fruit development than for 2019 and 2020, and higher temperature for the harvest period were observed, which may impact on berry physicochemical parameters. In fact, a clear distinction was observed in ASCA score plot between berries from the three harvests (Figure 3a). Samples are positioned in the order 2019–2018–2020 along the PC1, and the samples from 2018 are also displaced along the PC2. The difference between harvests along the PC1 is related to the total phenolics and titratable acidity content, with higher temperature in 2020 producing berries with higher phenolic content, and lower temperatures in 2019 producing berries with higher titratable acidity. A combination of high spring precipitation and hot summer in 2018 resulted in the sweeter berries with higher pH and °Brix (Table 5, and Figures S2 and S3). These results confirmed that climatic conditions, especially the water status, have a strong impact on elderberry chemical composition [27].

Figure 3b shows that the samples collected at the three fields were distributed along PC1, which explains 97% of the data variability. The order in which samples from each field are located corresponds to the increase in total phenolics, titratable acidity, antioxidant activity, and °Brix from the C3 to C1 (Table 5, and Figures S2 and S3). The highest values of these parameters observed for the berries collected at the field C1 may be attributed to the higher water availability at C1, which is located close to a river and irrigated twice a year. The water status associated with the irrigation and/or climatic conditions seems to be a relevant factor in modulating the berries’ composition, including sugars, phenolic composition, and acidity [27]. The field C3 has a higher planting density than the others, which is not recommended for this type of crop and that may have detrimental effect on the berry quality.

Cultivar was found to have lower impact on the physicochemical characteristics of ripe elderberries compared to the harvest year and field **(**Table 5 and Figures S2 and S3). Figure 3c shows that the cultivars ‘Sabugueira’, ‘Sabugueiro’, and ‘Bastardeira’ are dispersed along PC1, which explains 96% of the dataset variability. Some overlap is observed, especially between ‘Sabugueira’ and ‘Sabugueiro’ cultivars, unveiling higher similarity between both, which had higher phenolic content, antioxidant activity, titratable acidity, and °Brix compared to ‘Bastardeira’.

Interactions of all three factors were found statistically significant (Table 4). Interpretation of the interactions shown as biplots in Figure 3d–f is more complicated than the main effects (Figure 3a–c). The interaction of harvest year and field accounted for the second highest percentage of the dataset variance (19.3%), indicating the importance of the combined impact of the macro- and mesoclimate conditions on plant metabolism. Resulting complex metabolism alterations, in turn, affect the chemical composition of elderberries. In fact, the geoclimatic conditions [43] and location, environmental conditions, and plant nutrition, among others [44], have been reported as factors that modulate the plant metabolism and berry composition.

### 3.2. Evaluation of the Nutritional Value of Elderberry Juice and Respective Pomace Powder

**Figure 4:**
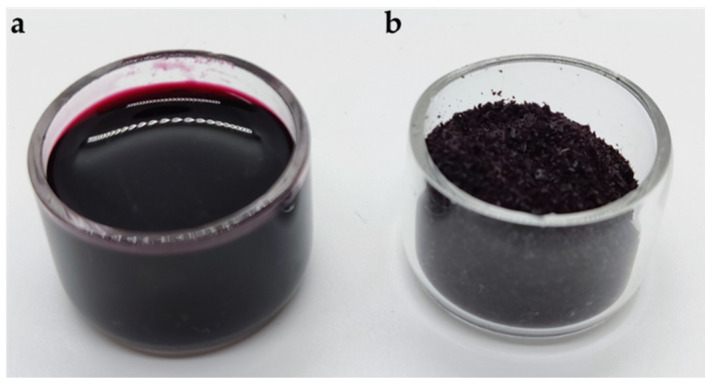
Products obtained from ripe elderberries. (**a**) Juice and respective (**b**) pomace powder.

The processing of fresh berries results in approximately 55% w/v of juice (Figure 4a), and 45% w/w of wet pomace, which corresponds a 13% w/w of dried pomace powder (Figure 4b). Table 6 presents the proximate composition of juice and dried pomace, which includes moisture, ash, lipid, protein, and carbohydrate contents. These food components may be of interest in the food industry for product development, quality control assessment, or regulatory purposes. The energetic value, mineral, and vitamin B6 composition were also determined.

Total carbohydrates are the main macronutrient of juice and dried pomace and represent 11.9 g/100 mL and 82.4 g/100 g dw, respectively. Carbohydrates, including fiber, are usually the principal component of the fruit-based products. In these cases, fiber content represents 0.5 g/100 mL of juice and 22.4 g/100 g of dry weight (dw) powder, showing that the powder is a significant source of dietary fiber. The fat content was relatively low for both products (<0.1 g/100 mL and 2.5 g/100 g dw), with polyunsaturated fat acids representing 1.4 g/100 g of powder. Ash content corresponds to 1.13 g/100 mL (juice) and 4.23 g/100 g dw (powder). Protein was found in levels of 0.8 g/100 mL in juice and 5.9 g/100 g dw in powder. The protein content of the elderberry is within the values reported for other berry pomaces, such as blackcurrant, redcurrant, gooseberry, rowanberry, and chokeberry, which range between 5.9 and 15.7 g per 100 g [45]. However, elderberry pomace exhibited lower fiber and fat contents than those reported for those berry pomaces, which ranged from 56.6 to 61 g per 100 g and 3.6 to 20.2 g per 100 g, respectively [45].

The juice has a caloric value of 50 kcal/100 mL, and 331 kcal/100 g was determined for the powder. The caloric value of the elderberry juice is similar to those reported for other natural juices widely consumed around the world, for instance, apple and orange juices [46,47].

Regarding the hydrophilic vitamin B6 content (Table 6), it may be pointed out that there is a significant amount of vitamin B6 in juice (0.27 mg/100 mL). A typical glass of juice (200 mL) allows an intake of up to 39% of the DRV of vitamin B6. This vitamin is involved in various biological responses; therefore, its deficiency is associated with the development of inflammatory diseases, such as allergy and rheumatoid arthritis, neuronal dysfunction, and risk of cardiovascular disease [48,49,50,51,52]. Epidemiological studies underlined the beneficial effects of vitamin B6 daily intake on the reduction in the incidence of cardiovascular diseases [53].

Four essential minerals, namely calcium, magnesium, iron, and selenium were detected in juice and dried pomace (Table 6), and, notably, magnesium and calcium contents were observed that are in accordance with the significant content of these minerals reported for fresh elderberries [24]. The elderberry juice contains 32.5 mg Mg/100 mL, 16.5 mg Ca/100 mL, while Fe and Se are present at lower amounts (0.11 mg and 0.78 µg per 100 mL, respectively). The consumption of this juice may promote the daily intake of these micronutrients, especially Mg and Ca. For instance, a glass of juice (200 mL) permits an intake of up to 17% of the DRV of magnesium. Comparison with other berry juices also allows the considerable nutritional value of elderberry juice to be inferred. In fact, the mineral content determined in a set of juices produced from red raspberry, black raspberry, blackcurrant, redcurrant, and bilberry unveiled that Mg content ranged between 5.31 and 12.69 mg/100 g, Ca ranged between 25.09 and 64.24 mg/100 g, and Fe ranged between 0.03 and 0.19 mg/100 g [55]. Moreover, the orange juice contains a lower concentration of these micronutrients compared to the elderberry juice, namely Ca (11 mg/100 g), Mg (9.5 mg/100 g), and Fe (0.2 mg/100 g) [47]. These results indicate that elderberry juice can be used as a source of essential minerals, which can contribute to preventing various diseases, namely arthritis, atherosclerosis, and diabetes, among others [56,57].

The Mg plays important roles in the physiological function of basically every organ, including heart, brain, bone, blood, and skeletal muscle. Moreover, the Mg is the most abundant intracellular cation and is involved in several metabolic and cellular processes in the body, and its deficiency has been associated with the risk of chronic diseases [6,58]. The daily intake of Ca is of major importance as this mineral represents the principal nutrient in the human body, stored in teeth and bone tissue, where it plays a key role in skeletal mineralization, regulation of hormonal secretion, transmission of nerve impulses, and vascular activities [6,59].

Despite elderberry juice and dry pomace possibly presenting a low contribution to the daily Fe and Se requirements, their role must be taken into consideration. In fact, Fe plays a crucial role in several functions, including oxygen transport, DNA synthesis and repair, electron transport, myelin formation maintenance, and for the synthesis of neurotransmitters [6,60]. Se is associated to a wide variety of physiological processes, including the central nervous system, male reproductive system, endocrine system, muscle function, cardiovascular system, and regulating immune cell functions [6,61].

## 4. Conclusions

The *Sambucus nigra* L. intra-variability among cultivars, ‘Sabugueiro’, ‘Sabugueira’, and ‘Bastardeira’, through three consecutive harvesting periods and fields unveiled that the harvest year, followed by field and the interaction of harvest × field, accounted for the highest impact on the berry’s physicochemical parameters. These results indicate the huge importance of the combined impact of the macro- and mesoclimate conditions on plant metabolism, rather than the cultivar. It is also important to point out the relevant effect of the soil water status, as the berries collected from the field with superior water accessibility exhibited the highest phenolics, titratable acidity, antioxidant activity, and °Brix.

Juice and dried pomace are good sources of carbohydrates, calcium, and magnesium and have low amounts of fat, making them low-energy foods. Dried pomace may also represent a potential alternative source of vegetal protein and fiber. Meanwhile consumption of juice may contribute significatively for the intake of vitamin B6, a vitamin involved in various biological responses. This integrated evaluation of the products obtained from elderberry is essential to define sustainable strategies for valorization of this natural product.

Finally, the strategy applied on the present study that includes a comprehensive assessment from crop biodiversity to nutritional value of juice and dried pomace allowed valuable information to be extracted that may support producer decisions and predict future agricultural productions of this crop. In fact, sustainable crop management can help to expand diet diversity while protecting the natural resources.

## Figures and Tables

**Table 1:** Main characteristics of the fields where the *Sambucus nigra* L. cultivars were collected.

Fields Characteristics					
Fields	Designation	Altitude (m)	Lines Orientation	Irrigation	Plant Age
Varosa	C1	260	North–South	2 times/year	7 years
Valverde	C2	640–700	East–West	No	7 years
São João de Tarouca	C3	570	East–West	No	5 years

**Table 2:** Physicochemical parameters of *Sambucus nigra* L. elderberry cultivars ‘Sabugueira’, ‘Sabugueiro’, and ‘Bastardeira’ from three consecutive harvesting years (2018–2020) and three fields, harvested at three maturity stages.

Cultivar	Field	Harvest ^a^	TSS (°Brix)			TA (g Citric Acid/L Juice)			pH		
			2018	2019	2020	2018	2019	2020	2018	2019	2020
‘Sabugueira’	C1	EM	8.00 ± 0.00	9.10 ± 0.20	9.80 ± 0.40	0.80 ± 0.01	1.08 ± 0.01	0.57 ± 0.01	3.82 ± 0.01	3.78 ± 0.03	4.10 ± 0.00
		AM	20.40 ± 0.80	14.70 ± 0.24	11.80 ± 0.40	0.54 ± 0.01	0.57 ± 0.01	0.56 ± 0.01	4.89 ± 0.01	4.31 ± 0.00	4.43 ± 0.00
		FM	29.60 ± 0.80	16.00 ± 0.00	12.60 ± 0.50	0.76 ± 0.01	0.62 ± 0.02	0.53 ± 0.01	4.89 ± 0.01	4.59 ± 0.01	4.57 ± 0.01
	C2	EM	9.10 ± 0.00	9.10 ± 0.20	8.20 ± 0.20	0.82 ± 0.01	1.16 ± 0.03	1.04 ± 0.03	4.05 ± 0.01	3.66 ± 0.01	3.59 ± 0.00
		AM	18.00 ± 0.00	10.0 ± 0.00	10.00 ± 0.00	0.49 ± 0.02	1.14 ± 0.02	0.50 ± 0.01	4.77 ± 0.01	3.61 ± 0.00	4.45 ± 0.02
		FM	13.20 ± 1.00	13.8 ± 0.40	17.40 ± 0.80	0.43 ± 0.01	0.69 ± 0.02	0.49 ± 0.01	4.60 ± 0.01	4.29 ± 0.01	4.85 ± 0.00
	C3	EM	10.00 ± 0.00	7.00 ± 0.00	9.20 ± 0.20	0.87 ± 0.01	1.29 ± 0.03	1.17 ± 0.02	3.93 ± 0.00	3.52 ± 0.02	3.43 ± 0.00
		AM	12.00 ± 0.00	12.00 ± 0.00	12.00 ± 0.00	0.49 ± 0.01	0.50 ± 0.01	0.43 ± 0.01	4.47 ± 0.01	4.25 ± 0.02	4.63 ± 0.01
		FM	17.60 ± 0.80	13.40 ± 0.49	14.50 ± 0.40	0.38 ± 0.01	0.74 ± 0.04	0.40 ± 0.01	4.90 ± 0.01	4.13 ± 0.01	4.66 ± 0.01
‘Sabugueiro’	C1	EM	8.00 ± 0.00	7.00 ± 0.00	9.00 ± 0.00	0.74 ± 0.02	1.09 ± 0.01	0.65 ± 0.02	4.22 ± 0.00	4.10 ± 0.01	3.85 ± 0.00
		AM	19.40 ± 0.50	15.00 ± 0.00	11.10 ± 0.20	0.49 ± 0.01	0.57 ± 0.01	0.51 ± 0.01	5.03 ± 0.01	4.51 ± 0.02	4.11 ± 0.01
		FM	16.00 ± 0.00	14.80 ± 0.40	11.80 ± 0.40	0.57 ± 0.01	0.61 ± 0.05	0.50 ± 0.01	4.84 ± 0.01	4.73 ± 0.01	4.34 ± 0.00
	C2	EM	7.50 ± 0.00	9.30 ± 0.24	8.50 ± 0.40	1.02 ± 0.02	1.12 ± 0.09	0.71 ± 0.02	3.98 ± 0.02	3.81 ± 0.02	3.75 ± 0.00
		AM	15.60 ± 0.80	10.40 ± 0.80	14.00 ± 0.00	0.74 ± 0.01	0.68 ± 0.01	0.67 ± 0.03	4.43 ± 0.01	4.20 ± 0.02	4.74 ± 0.01
		FM	14.80 ± 1.00	12.00 ± 0.00	14.40 ± 0.50	0.49 ± 0.01	0.76 ± 0.01	0.45 ± 0.01	4.59 ± 0.01	4.42 ± 0.02	4.94 ± 0.01
	C3	EM	10.00 ± 0.00	5.80 ± 0.24	7.40 ± 0.4	0.45 ± 0.01	0.95 ± 0.05	0.72 ± 0.01	4.05 ± 0.00	3.99 ± 0.02	3.97 ± 0.00
		AM	13.60 ± 0.50	10.60 ± 0.20	10.00 ± 0.00	0.50 ± 0.01	1.10 ± 0.08	0.63 ± 0.02	4.45 ± 0.01	3.98 ± 0.01	4.61 ± 0.00
		FM	13.60 ± 0.80	11.60 ± 0.49	12.90 ± 0.20	0.34 ± 0.02	0.64 ± 0.01	0.29 ± 0.01	4.93 ± 0.02	4.10 ± 0.01	4.67 ± 0.01
‘Bastardeira’	C1	EM	8.00 ± 0.00	9.00 ± 0.00	7.80 ± 0.20	0.90 ± 0.03	1.13 ± 0.04	0.66 ± 0.03	3.96 ± 0.01	3.94 ± 0.03	3.90 ± 0.00
		AM	15.40 ± 0.50	12.40 ± 0.20	10.10 ± 0.20	0.44 ± 0.01	0.56 ± 0.01	0.49 ± 0.01	4.75 ± 0.00	4.29 ± 0.02	4.18 ± 0.00
		FM	16.40 ± 0.80	14.00 ± 0.00	11.80 ± 0.40	0.35 ± 0.01	0.47 ± 0.01	0.48 ± 0.01	5.00 ± 0.01	4.61 ± 0.01	4.35 ± 0.02
	C2	EM	9.70 ± 0.20	10.10 ± 0.20	9.60 ± 0.40	0.94 ± 0.02	1.33 ± 0.01	0.73 ± 0.02	3.80 ± 0.00	3.96 ± 0.00	3.79 ± 0.00
		AM	20.00 ± 0.00	10.60 ± 0.37	11.80 ± 0.40	0.42 ± 0.01	0.70 ± 0.01	0.45 ± 0.01	4.92 ± 0.01	4.11 ± 0.02	4.58 ± 0.00
		FM	17.20 ± 1.00	16.00 ± 0.00	13.80 ± 0.40	0.33 ± 0.01	0.83 ± 0.01	0.50 ± 0.01	4.81 ± 0.01	4.41 ± 0.01	4.72 ± 0.00
	C3	EM	8.50 ± 0.00	5.00 ± 0.00	7.10 ± 0.20	0.62 ± 0.02	1.00 ± 0.01	0.83 ± 0.01	3.71 ± 0.01	3.74 ± 0.02	3.51 ± 0.00
		AM	10.80 ± 0.40	8.00 ± 0.00	11.30 ± 0.40	0.46 ± 0.01	1.19 ± 0.01	0.66 ± 0.03	4.43 ± 0.00	3.68 ± 0.03	4.29 ± 0.01
		FM	16.00 ± 0.00	8.80 ± 0.00	12.90 ± 0.20	0.35 ± 0.01	0.86 ± 0.03	0.33 ± 0.01	5.02 ± 0.01	4.01 ± 0.01	4.56 ± 0.00

Values expressed as mean ± standard deviation (*n* = 5); ^a^ EM (containing berries at early stage of maturity—approximately 30% of berries exhibited the final color); AM (containing almost mature berries—approximately 70% of berries exhibited the final color); and FM (containing fully mature berries).

**Table 3:** Total phenolic content (TPC) and antioxidant activity of *Sambucus nigra* L. elderberry cultivars ‘Sabugueira’, ‘Sabugueiro’, and ‘Bastardeira’ from three consecutive harvesting years (2018–2020) and three fields, harvested at three maturity stages.

Cultivar	Field	Harvest ^a^	TPC (g GAE/L Juice) ^b^			Antioxidant Activity (mmol TE/L Juice) ^c^		
			2018	2019	2020	2018	2019	2020
‘Sabugueira’	C1	EM	1.35 ± 0.07	4.07 ± 0.11	7.91 ± 0.28	3.25 ± 0.25	22.08 ± 0.50	21.44 ± 0.15
		AM	14.61 ± 0.27	10.44 ± 0.11	12.49 ± 0.12	73.90 ± 3.25	83.68 ± 0.80	38.17 ± 0.79
		FM	19.12 ± 1.14	14.01 ± 0.30	19.22 ± 0.21	80.20 ± 4.06	84.77 ± 4.26	87.81 ± 0.19
	C2	EM	2.31 ± 0.16	4.07 ± 0.11	4.15 ± 0.04	9.13 ± 0.47	20.83 ± 0.34	10.40 ± 0.26
		AM	8.07 ± 0.42	7.01 ± 0.15	22.36 ± 0.17	50.58 ± 4.56	48.91 ± 0.93	70.67 ± 0.33
		FM	8.52 ± 0.43	10.27 ± 0.33	22.37 ± 0.29	50.76 ± 4.18	49.03 ± 2.02	78.46 ± 0.56
	C3	EM	1.77 ± 0.17	1.89 ± 0.07	5.35 ± 0.07	3.40 ± 0.27	3.53 ± 0.17	8.68 ± 0.23
		AM	8.52 ± 0.26	6.42 ± 0.15	12.34 ± 0.33	20.11 ± 0.87	38.63 ± 1.60	43.67 ± 0.41
		FM	8.65 ± 0.50	7.41 ± 0.26	17.71 ± 0.31	40.09 ± 2.82	32.67 ± 1.41	55.50 ± 0.33
‘Sabugueiro’	C1	EM	3.94 ± 0.26	4.16 ± 0.03	5.98 ± 0.20	12.77 ± 0.44	16.46 ± 0.72	17.48 ± 0.85
		AM	9.30 ± 0.47	11.73 ± 0.06	13.08 ± 0.32	56.59 ± 2.00	97.57 ± 5.15	37.53 ± 0.78
		FM	14.96 ± 0.64	16.05 ± 0.51	19.16 ± 0.30	78.76 ± 2.57	101.48 ± 2.01	81.43 ± 0.84
	C2	EM	2.78 ± 0.17	4.91 ± 0.21	6.88 ± 0.26	8.92 ± 0.55	26.17 ± 0.41	18.05 ± 0.45
		AM	10.96 ± 0.25	9.38 ± 0.29	18.96 ± 0.60	64.83 ± 3.97	70.80 ± 2.39	64.14 ± 1.07
		FM	10.00 ± 0.53	12.88 ± 0.51	19.50 ± 0.48	50.02 ± 4.41	62.57 ± 1.49	66.49 ± 0.21
	C3	EM	3.63 ± 7.85	2.50 ± 0.02	4.95 ± 0.14	13.01 ± 0.51	6.16 ± 0.23	14.21 ± 0.38
		AM	7.85 ± 0.84	5.01 ± 0.13	11.44 ± 0.35	51.24 ± 3.97	26.67 ± 0.82	43.61 ± 0.53
		FM	12.40 ± 0.60	8.09 ± 0.14	18.05 ± 0.41	65.76 ± 3.78	35.80 ± 0.52	55.71 ± 0.50
‘Bastardeira’	C1	EM	2.17 ± 0.08	3.82 ± 0.09	5.33 ± 0.16	5.53 ± 0.46	16.84 ± 0.96	12.75 ± 0.35
		AM	6.46 ± 0.34	7.28 ± 0.31	11.13 ± 0.12	35.95 ± 3.54	54.49 ± 1.25	32.81 ± 0.31
		FM	9.47 ± 0.09	9.34 ± 0.34	17.58 ± 0.71	48.25 ± 1.68	64.22 ± 1.38	72.75 ± 1.75
	C2	EM	2.47 ± 0.14	6.97 ± 0.11	6.42 ± 0.24	10.39 ± 0.59	32.22 ± 0.20	19.57 ± 0.25
		AM	9.75 ± 0.49	8.94 ± 0.29	17.99 ± 0.66	50.29 ± 2.93	64.09 ± 2.77	64.14 ± 0.91
		FM	8.08 ± 0.29	14.25 ± 0.26	21.06 ± 0.66	41.86 ± 2.47	69.79 ± 1.30	69.08 ± 0.95
	C3	EM	2.51 ± 0.16	1.54 ± 0.07	3.76 ± 0.11	10.55 ± 0.38	3.93 ± 0.27	10.04 ± 0.22
		AM	7.62 ± 0.55	3.46 ± 0.14	10.82 ± 0.84	42.98 ± 0.77	18.52 ± 0.55	41.34 ± 0.29
		FM	11.55 ± 0.51	7.29 ± 0.14	13.39 ± 0.23	62.26 ± 1.92	34.38 ± 1.19	49.09 ± 0.26

Values are expressed as mean ± standard deviation (*n* = 5). ^a^ EM (containing berries at early stage of maturity—approximately 30% of berries exhibited the final color); AM (containing almost mature berries—approximately 70% of berries exhibited the final color); and FM (containing fully mature berries); ^b^ gallic acid equivalent per liter of juice; ^c^ mmol equivalent Trolox per liter of juice.

**Table 4:** Significance testing of factors harvest, field, and cultivar for *Sambucus nigra* L., determined at ripe berries.

Factors	*p*-Value (2000 Permutations)	Explained Variance (%)
Harvest	<0.0005	35.1
Field	<0.0005	18.3
Cultivar	0.003	5.7
Interactions		
Harvest × Field	<0.0005	19.3
Field × Cultivar	0.015	6.5
Harvest × Cultivar	<0.0005	5.6

For *p* < 0.05, the differences were considered significant.

**Table 5:** Descriptive statistics of physicochemical parameters obtained from ripe berries from *Sambucus nigra* L., collected at three consecutive harvests (2018–2020), three fields, and cultivars ‘Sabugueira (Sa)’, ‘Sabugueiro (So)’, and ‘Bastardeira (B)’.

	2018 ^a^	2019 ^a^	2020 ^a^	C1 ^b^	C2 ^b^	C3 ^b^	Sa ^c^	So ^c^	B ^c^
**TSS (°Brix)**									
Median	16.00	14.00	13.00	15.00	14.00	13.00	14.50	14.00	14.00
Minimum	12.00	8.00	11.00	11.00	12.00	8.00	12.00	11.00	8.00
Maximum	30.00	16.00	18.00	30.00	18.00	18.00	30.00	16.00	18.00
25% Percentile	14.00	12.00	12.00	12.00	14.00	12.25	13.50	12.00	12.75
75% Percentile	18.00	15.00	14.00	16.00	16.00	15.00	18.00	15.00	16.00
**Titratable acidity (g citric acid/L juice)**									
Median	0.38	0.59	0.48	0.52	0.48	0.37	0.49	0.50	0.41
Minimum	0.32	0.35	0.27	0.34	0.32	0.27	0.35	0.27	0.32
Maximum	0.76	0.78	0.54	0.76	0.78	0.65	0.76	0.78	0.50
25% Percentile	0.34	0.43	0.40	0.48	0.42	0.33	0.40	0.45	0.34
75% Percentile	0.48	0.65	0.50	0.59	0.51	0.41	0.61	0.62	0.48
**pH**									
Median	4.87	4.40	4.65	4.61	4.60	4.65	4.60	4.66	4.61
Minimum	4.57	4.00	4.33	4.33	4.28	4.00	4.11	4.09	4.00
Maximum	5.05	4.74	4.96	5.01	4.96	5.05	4.91	4.96	5.05
25% Percentile	4.81	4.12	4.56	4.52	4.42	4.12	4.56	4.40	4.40
75% Percentile	4.96	4.59	4.73	4.84	4.82	4.91	4.85	4.86	4.82
**TPC (g GAE/L juice)**									
Median	9.84	10.44	18.88	16.14	12.92	11.43	14.01	14.89	11.43
Minimum	7.71	7.03	13.00	8.86	7.82	7.03	7.03	7.87	7.08
Maximum	20.21	16.62	22.71	20.21	22.71	18.55	22.71	20.26	21.65
25% Percentile	8.97	7.94	17.77	13.68	9.57	7.79	8.97	11.67	8.94
75% Percentile	12.93	14.09	19.68	18.92	19.68	13.60	19.24	18.41	14.50
**Antioxidant activity (mmol TE/L juice)**									
Median	54.04	63.04	68.82	80.28	63.36	49.18	55.50	66.38	62.47
Minimum	36.48	30.95	48.73	45.71	39.06	30.95	30.95	35.04	32.49
Maximum	83.54	103.0	88.02	103.0	79.17	68.04	90.55	103.0	74.84
25% Percentile	44.58	35.61	55.72	70.95	49.68	35.61	44.47	55.58	46.29
75% Percentile	67.91	71.35	79.10	87.24	68.83	56.23	81.88	80.05	68.83

^a^ corresponds to a median of 9 samples (3 fields and 3 cultivars, with 5 replicates per sample; *n* = 45) harvested per year; ^b^ corresponds to a median of 9 samples (3 cultivars and 3 harvest, with 5 replicates per sample; *n* = 45) per field; ^c^ corresponds to a median of 9 samples (3 fields and 3 harvest, with 5 replicates per sample; *n* = 45) per cultivar.

**Table 6:** Proximate composition, energetic value, mineral, and vitaminic composition of *Sambucus nigra* L. juice and dried pomace powder. Dietary reference values (DRV) are also reported.

Nutritional Information	Juice	Pomace Powder	DRV [54]
	Per 100 mL	Per 100 g	
**Energy (kJ/kcal)**	211/50	1391/331	8400/2000
**Fat (g)**	<0.1 (LQ)	2.5	70
Saturated fat acids (g)	<0.0001 (LQ)	0.6	20
Monounsaturated fat acids (g)	- *	0.3	-
Polyunsaturated fat acids (g)	-	1.4	-
*Trans* Fat acids (g)	-	<0.0001 (LQ)	-
**Total carbohydrates (g)**	11.9	82.4	260
**Total sugars (g)**	11.4	44.7	90
**Fiber (g)**	0.5	22.4	30
**Total protein (g)**	0.8	5.9	50
**Vitamins**			
Vitamin B6 (mg)	0.27	-	1.4
**Minerals**			
Calcium (mg)	16.5	164	800
Magnesium (mg)	32.5	183	375
Iron (mg)	0.11	2.41	14
Selenium (μg)	0.78	4.2	55
**Salt** **(Na × 2.5) (g)**	<0.00625 (LQ)	<0.0206 (LQ)	6
**Water (g)**	89.4	5.0	-
**Ash (g)**	1.13	4.23	-

* Not determined in samples; LQ—limit of quantification.

## Data Availability

The data supporting the findings of this study are included within the article.

## Supplementary Materials

The following are available online at https://www.mdpi.com/article/10.3390/foods11010104/s1, Figure S1: Dispersion of the (a) total phenolic, and (b) antioxidant activity values of all elderberries under study according to the respective TSS expressed as °Brix, showing in each graphic, a positive association between the levels of variation of both parameters through ripening. Figure S2: Descriptive statistics of physicochemical content of ripe berries from Sambucus nigra L., from three consecutive harvests (2018–2020), three fileds (C1, C2 and C3) and cultivars ‘Sabugueira’, ‘Sabugueiro’ and ‘Bastardeira’. Bars represent 25th and 75th percentiles with median in middle. The red line above and below represents the maximum and minimum, respectively. Results for TSS expressed as °Brix, and titratable acidity expressed as g citric acid/L juice. Figure S3: Descriptive statistics of total phenolic content and antioxidant activity reported ripe berries from Sambucus nigra L., from three consecutive harvests (2018–2020), three fileds (C1, C2 and C3) and cultivars ‘Sabugueira’, ‘Sabugueiro’ and ‘Bastardeira’. Bars represent 25th and 75th percentiles with median in middle. The red line above and below represents the maximum and minimum, respectively. Results for total phenolic compounds expressed as g GAE/L juice, and antioxidant activity expressed as mmol TE/L juice.
