# Supplementary material for: Sustainable Valorization of Sambucus nigra L. Berries: From Crop Biodiversity to Nutritional Value of Juice and Pomace
Source: Foods. 2021 Dec 31;11(1):104. doi: 10.3390/foods11010104 (PMC8750068; doi:10.3390/foods11010104)
Supplement: Supplementary file 1 [file foods-11-00104-s001.zip › Supplementary Information.pdf]

foods

Sustainable valorization of *Sambucus nigra* L. berries: from crop biodiversity to nutritional value of juice and respective pomace

Carina Pedrosa Costa [1], Samuel Patinha [2], Alisa Rudnitskaya [3], Sónia A. O. Santos [2], Armando J. D. Silvestre [2] and Sílvia M. Rocha [1,*]

[1] LAQV-REQUIMTE & Department of Chemistry, Campus Universitário Santiago, University of Aveiro, 3810-193 Aveiro, Portugal; carina.pedrosa@ua.pt
[2] CICECO-Aveiro Institute of Materials & Department of Chemistry, Campus Universitário Santiago, University of Aveiro, 3810-193 Aveiro, Portugal; jsamuelpatinha@ua.pt (S.P.); santos.sonia@ua.pt (S.A.O.A.); armsil@ua.pt (A.J.D.S.)
[3] CESAM & Department of Chemistry, University of Aveiro, Campus Universitário Santiago, 3810-193 Aveiro, Portugal; alisa@ua.pt
* Correspondence: smrocha@ua.pt; Tel.: +351-234-401524

Supplementary Information

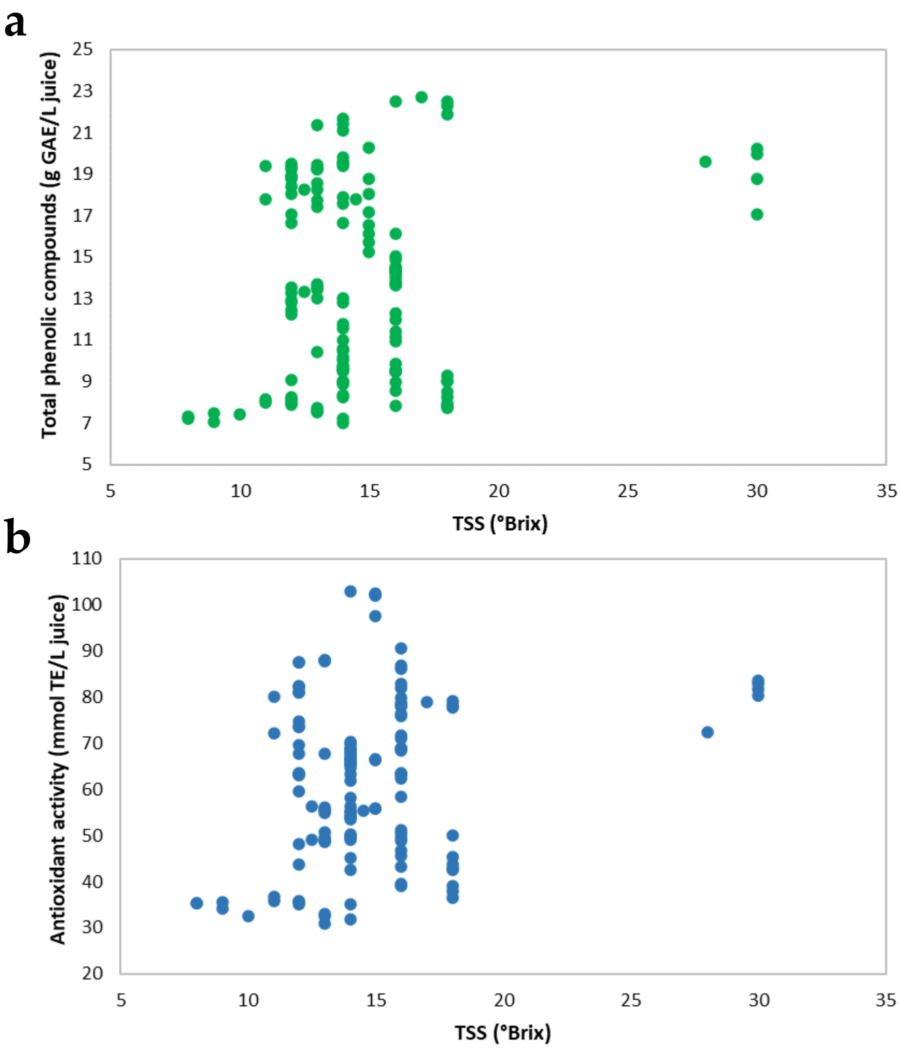

Figure S1. Dispersion of the (**a**) total phenolic, and (**b**) antioxidant activity values of all elderberries under study according to the respective TSS expressed as °Brix, showing, in each graphic, a positive association between the levels of variation of both parameters through ripening.

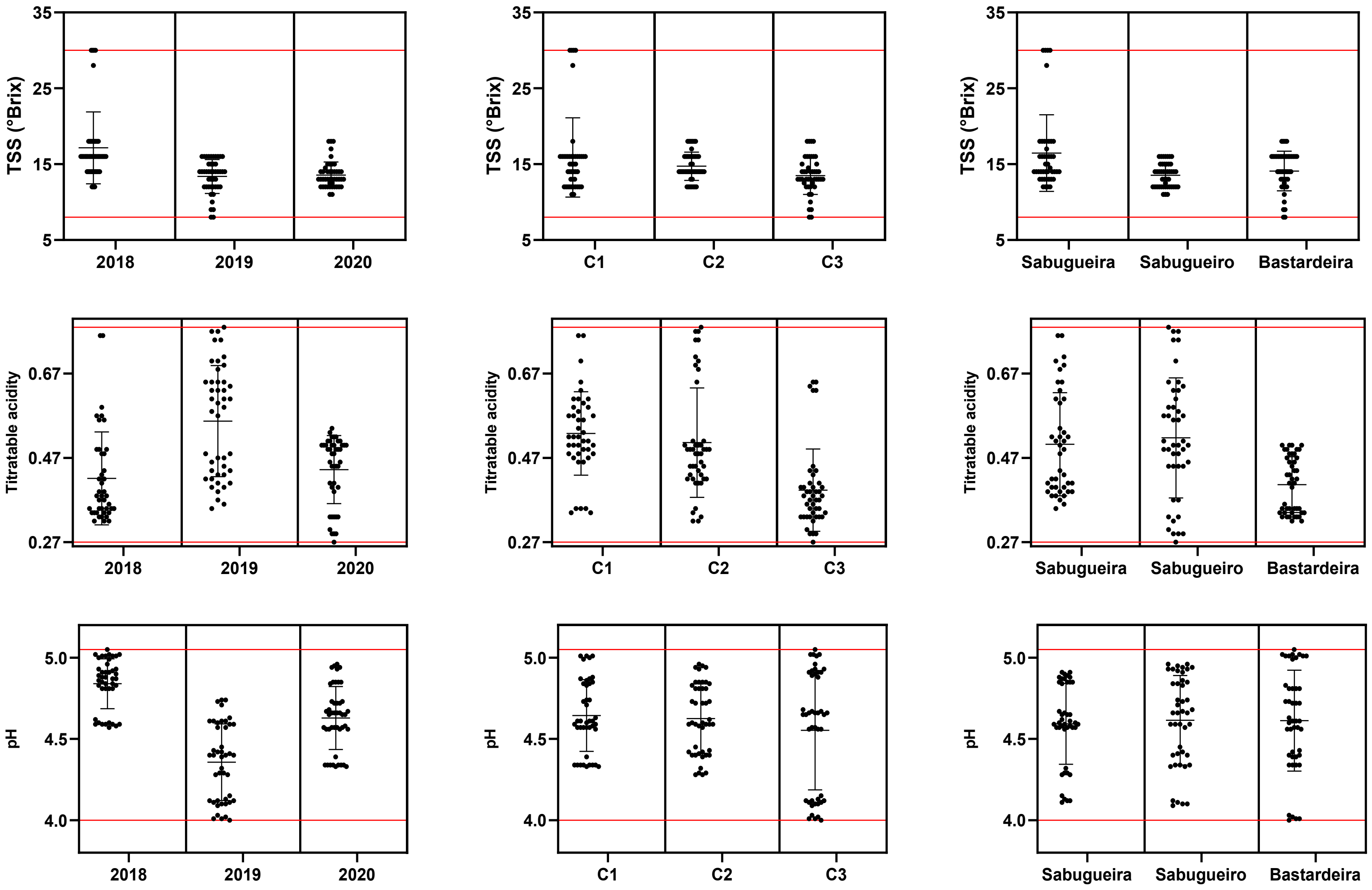

Figure S2. Descriptive statistics of physicochemical content of ripe berries from *Sambucus nigra* L., from three consecutive harvests (2018-2020), three fileds (C1, C2 and C3) and cultivars 'Sabugueira', 'Sabugueiro' and 'Bastardeira'. Bars represent 25th and 75th percentiles with median in middle. The red line above and below represents the maximum and minimum, respectively. Results for TSS expressed as °Brix, and titratable acidity expressed as g citric acid/L juice.

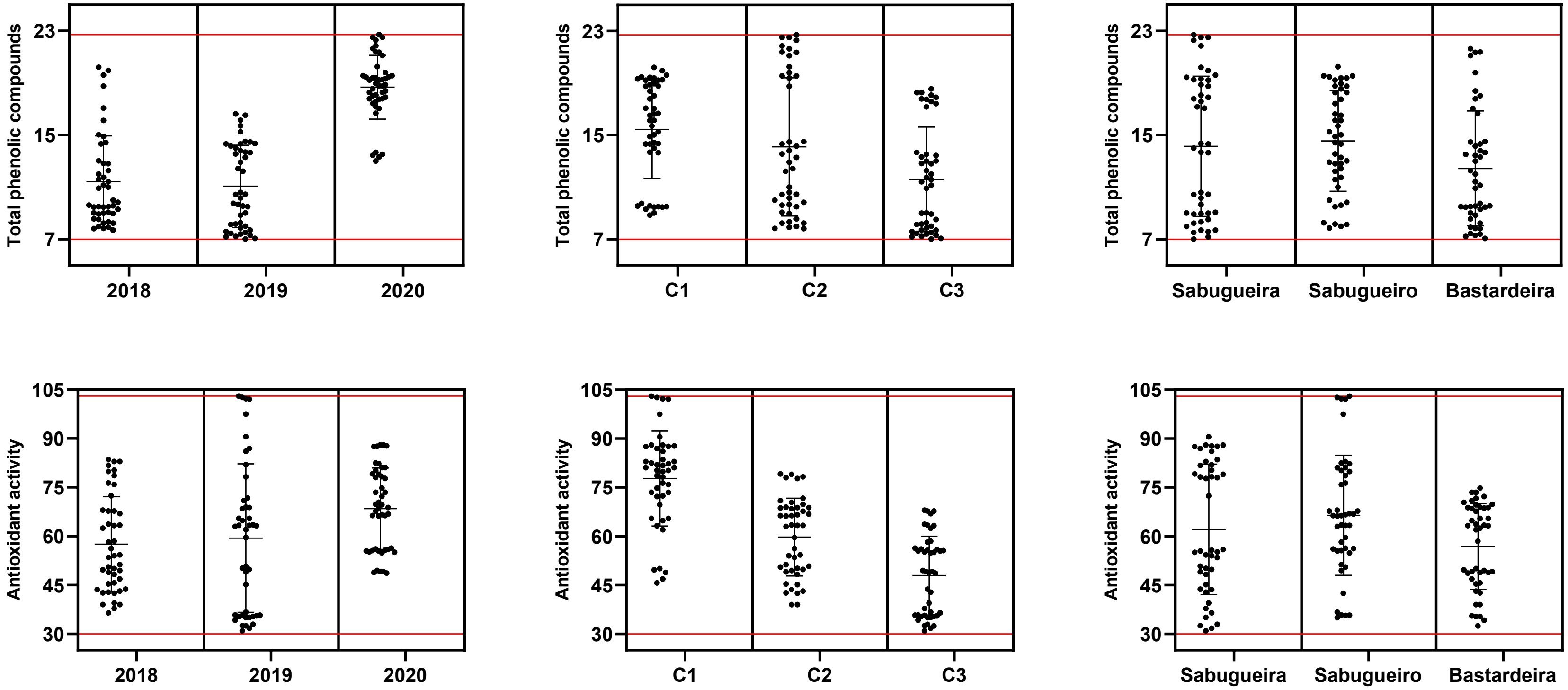

Figure S3. Descriptive statistics of total phenolic content and antioxidant activity reported ripe berries from *Sambucus nigra* L., from three consecutive harvests (2018-2020), three fileds (C1, C2 and C3) and cultivars 'Sabugueira', 'Sabugueiro' and 'Bastardeira'. Bars represent 25th and 75th percentiles with median in middle. The red line above and below represents the maximum and minimum, respectively. Results for total phenolic compounds expressed as g GAE/L juice, and antioxidant activity expressed as mmol TE/L juice.
